# Involvement of Phytochemical-Encapsulated Nanoparticles’ Interaction with Cellular Signalling in the Amelioration of Benign and Malignant Brain Tumours

**DOI:** 10.3390/molecules27113561

**Published:** 2022-06-01

**Authors:** Sidharth Mehan, Navneet Arora, Sonalika Bhalla, Andleeb Khan, Muneeb U Rehman, Badrah S. Alghamdi, Torki Al Zughaibi, Ghulam Md Ashraf

**Affiliations:** 1 Department of Pharmacology, Neuropharmacology Division, ISF College of Pharmacy, Moga 142001, India; sidharthmehan@isfcp.org (S.M.); sonalikabhalla97@gmail.com (S.B.); 2 Department of Pharmacy Practice, ISF College of Pharmacy, Moga 142001, India; idnavneetarora@gmail.com; 3 Department of Pharmacology and Toxicology, College of Pharmacy, Jazan University, Jazan 45142, Saudi Arabia; 4 Department of Clinical Pharmacy, College of Pharmacy, King Saud University, Riyadh 11451, Saudi Arabia; 5 Pre-Clinical Research Unit, King Fahd Medical Research Center, King Abdulaziz University, Jeddah 21589, Saudi Arabia; basalghamdi@kau.edu.sa (B.S.A.); ashraf.gm@gmail.com (G.M.A.); 6 Neuroscience Unit, Department of Physiology, Faculty of Medicine, King Abdulaziz University, Jeddah 21589, Saudi Arabia; 7 King Fahd Medical Research Center, King Abdulaziz University, Jeddah 21589, Saudi Arabia; taalzughaibi@kau.edu.sa; 8 Department of Medical Laboratory Sciences, Faculty of Applied Medical Sciences, King Abdulaziz University, Jeddah 21589, Saudi Arabia

**Keywords:** brain tumour, phytochemicals, nanoparticles, glioblastoma, astrocytoma, blood–brain barrier

## Abstract

Brain tumours have unresolved challenges that include delay prognosis and lower patient survival rate. The increased understanding of the molecular pathways underlying cancer progression has aided in developing various anticancer medications. Brain cancer is the most malignant and invasive type of cancer, with several subtypes. According to the WHO, they are classified as ependymal tumours, chordomas, gangliocytomas, medulloblastomas, oligodendroglial tumours, diffuse astrocytomas, and other astrocytic tumours on the basis of their heterogeneity and molecular mechanisms. The present study is based on the most recent research trends, emphasising glioblastoma cells classified as astrocytoma. Brain cancer treatment is hindered by the failure of drugs to cross the blood–brain barrier (BBB), which is highly impregnableto foreign molecule entry. Moreover, currently available medications frequently fail to cross the BBB, whereas chemotherapy and radiotherapy are too expensive to be afforded by an average incomeperson and have many associated side effects. When compared to our current understanding of molecularly targeted chemotherapeutic agents, it appears that investigating the efficacy of specific phytochemicals in cancer treatment may be beneficial. Plants and their derivatives are game changers because they are efficacious, affordable, environmentally friendly, faster, and less toxic for the treatment of benign and malignant tumours. Over the past few years, nanotechnology has made a steady progress in diagnosing and treating cancers, particularly brain tumours. This article discusses the effects of phytochemicals encapsulated in nanoparticles on molecular targets in brain tumours, along with their limitations and potential challenges.

## 1. Introduction

Brain tumours are serious, life-threatening, and incurable diseases that contribute significantly to human suffering and the economic burden of the healthcare system [1]. The central nervous system (CNS) has a complicated anatomy, making it more vulnerable to approximately 130 primary neoplasms [2]. Primary malignant brain tumours are the leading cause of death in children with solid tumours and the third leading cause of cancer-related death in adolescents and adults aged 15 to 34 [3,4].

GBM grade-IV astrocytoma is a brain tumour characterised by star-shaped glial cells. It is a rapidly spreading tumour that affects nearby brain areas, particularly cerebral hemispheres’ frontal and temporal lobes. GBM has no known cause, but hereditary diseases such asschwannomatosis and neurofibromatosis, which cause the tumour to grow in the nervous system, are significant risk factors [5]. Pilocytic astrocytomas are the most prevalent primary tumours in children and adolescents, accounting for 15.6 percent of all brain tumours and 5.4 percent of all gliomas. They are found in infratentorial regions across the brain, especially in the cerebellum and midline cerebral structures such as the optic nerve, hypothalamus, and brain stem [6]. Pilocytic astrocytomas are extremely rare, non-invasive, and surgically curable, whereas glioblastoma is the typical intraparenchymal adult tumour that is highly invasive and practically incurable [7]. GBM malignant tumours develop from glial cells in the brain and have a dismal prognosis, with a 5-year survival rate of approximately 56% [8]. GBM has a peak prevalence of 55–60 years, with males having a 1.6 times higher incidence ratio than females, and it can strike at any age [9].

Neuroblastomas (NBs) are another form of common primary CNS tumour. Theserare brain tumours primarily affect young children and areone of the leading causes of childhood cancer death [10]. The most common extracranial solid malignant tumours in neonates and children arise from primitive sympathetic ganglion cells and appear as an adrenal mass [11]. With a less than 50% survival rate, the NBs account for 15% of all cancer-related mortalities in children [12].

The incidence of brain tumourshas increased over the last few decades [13]. Despite significant advancements in cancertreatment, it remains one of the world’s leading causes of death, killing nearly 9.6 million people each year [14]. In addition to the genetic predisposition and environmental risk factors, increased oxidative stress has been identified as a possible common cause of brain tumours [15]. As our understanding of the molecular pathways behind cancer growth has evolved, anticancer medicines have been discovered. Chemically synthesised medications have not resulted in a significant increase in overall survival rates [16]. The most considerable impediment to therapeutic efficacy is the development of chemoresistance in cancer cells to conventional chemotherapy agents [17]. However, the side effects associated with conventional treatments include cardiotoxicity, nephrotoxicity, hepatotoxicity, neurotoxicity, myelosuppression, alopecia, mucositis, and gastrointestinal toxicity [18,19,20]. Due to the aggressive nature of glioblastoma, surgical treatment is nearly impossible and frequently futile. CNS tumours are treated with radiation, chemotherapy, and surgery, depending on the degree of malignancy (GBM and NB) [21]. While some currently available chemotherapeutic drugs strive to reduce the further worsening these CNS tumours by targeting the DNA of cancer cells, many have been linked to a variety of side effects in children, including endocrine deficits and chronic neurocognitive impairment [16]. Due to the limited availability of effective treatment options for brain tumours, clinicians must constantly search for new medications to assist their patients [22]. More therapeutic methods that can inhibit the growth of cancerous cells while having minimal side effects on healthy cells must be thoroughly investigated. According to the FDA, approximately 80% of all cancer treatment drugs in the last 30 years are either natural products themselves or their derivatives [16]. Multi-targeted agents that suppress survival and oncogenic pathways, either alone or in combination, to expose cancer cells to conventional therapies are crucial for overcoming drug resistance [23].

Several preclinical and clinical studies have demonstrated that phytochemicals have the potential to lower drug resistance and sensitise cancer cells to chemotherapeutic agents by interfering with proteins/multiple genes/pathways that control vital factors in tumour growth and progression, such as pro-apoptotic protein activation, anti-apoptotic protein inhibition, reduced expression of various transcription factors, and endocrine disruption [24]. Due to their broad range of biological activities, including anti-inflammation, antioxidation, anti-tumour, anti-mutagenesis, and immunomodulation, phytochemicals have a significant potential to eradicate the side effects associated with chemotherapy and radiotherapy [25]. The chemical diversity and biological properties of plants make them ideal for use as adjuvant therapy to mitigate the side effects of cancer therapy [26]. From even more than 3000 plants, many antitumour drugs have been studied, and many phytochemicals have been used to develop safe anticancer drugs or adjunctive therapies [27]. Many studies have shown that phytochemicals have an efficient and promising alternative therapeutic potential against tumour types, which demonstrates the aim to use phytochemicals as adjunct cancer targeted therapies to develop novel combinatorial cancer treatment strategies for the efficient and safe treatment of tumours [28]. Emerging in vitro and in vivo studies strongly suggest that these naturallyderived analogues and preparations may be much more effective in reducing the incidence and treating various cancers [29]. Cancer therapy protective effects have been shown to appear in tumour cells and animal cancer models, but some are in phase I and II clinical trials [30]. Plant-derived drugs camptothecin, vincristine, and paclitaxel have been effectively used to treat various cancers. In contrast to available conventional treatments, numerous compounds derived from plants, such as EGCG, curcumin, and genistein, have been widely examined for their possible use as adjuvant therapies for several tumours [31]. These phytochemicals frequently work by modulating molecular pathways, such as antioxidant levels, cell cycle arrest, enhanced carcinogen inactivation, apoptosis, immune system control, and proliferation suppression that contribute to prevention of cancer growth and progression [32,33]. These natural compounds have acritical role in cancer amelioration as they can modulate the tumour suppressor role or oncogenic non-coding and coding transcript expression [34]. It is now clear that phytochemical-rich diets can modulate cellular stress response pathways, playing a significant protective role in attenuating pro-inflammatory and oxidative harm [35]. However, since most phytochemicals have only been subjected to preclinical testing, further clinical trials are required to assess their therapeutic efficacy [36].

**Figure 1:**
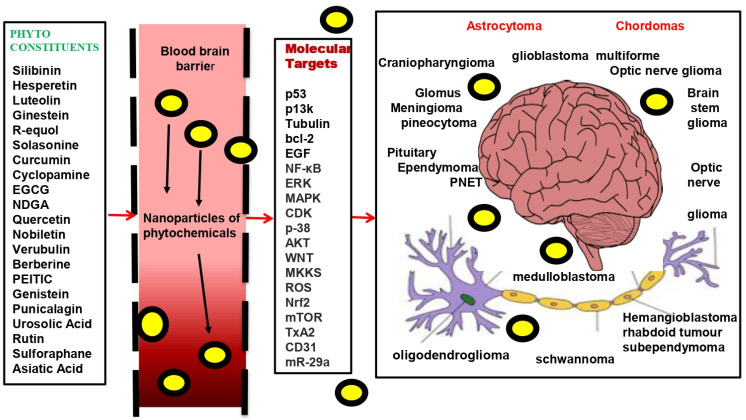
Phytochemical-encapsulated nanoparticle-based delivery to cross the BBB with increased absorption and stability to attack molecular targets in order to tackle various brain tumours. Phytochemicals that cannot cross the BBB are encapsulated in nanoparticles, which can improve the drug’s stability, absorption, and bioavailability. These nanoparticles modulate the molecular targets involved in different brain tumours (benign and malignant), as listed here.

Phytochemicals are restricted by their poor bioavailability, poor cell penetration, low aqueous solubility, hepatic disposition, rapid absorption by normal tissues, and limited therapeutic index, despite their excellent antitumor activity [37,38]. Polymeric nanotechnology drug delivery, which can increase bioavailability and absorption in the gastrointestinal system, as well astransfer to target organs, is a viable choice [39,40]. As a result, this segment focuses on the entire subject of brain tumours, their global importance and therapeutic challenges, and the use of nanoparticles in brain tumour treatment. This manuscript investigates the successful nano-based delivery method for phytochemicals to improve their bioavailability to prevent and treat brain tumours by attacking molecular targets, as evidenced by the several recent preclinical and clinical studies. The new approach of using nano-based delivery of phytochemicals as a sword to attack brain tumours most safely and cost-effectively is being investigated (Figure 1).

## 2. Natural Products/Phytochemicals against the Plethora of Brain Tumours

**Figure 2:**
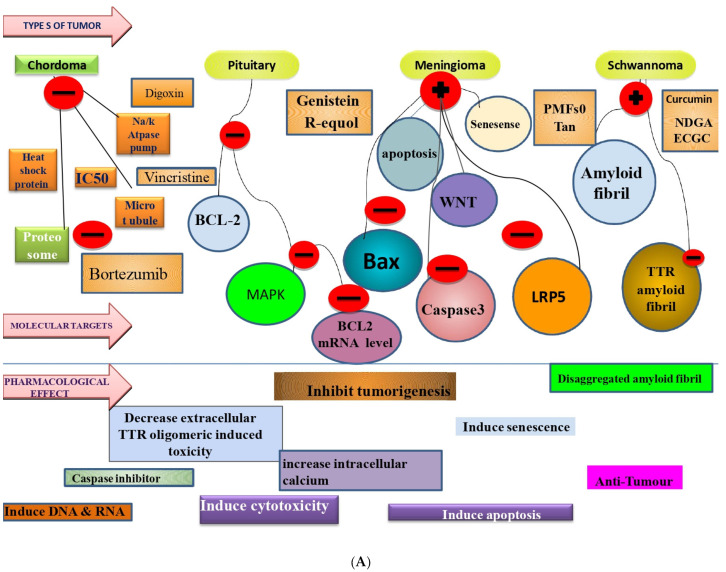

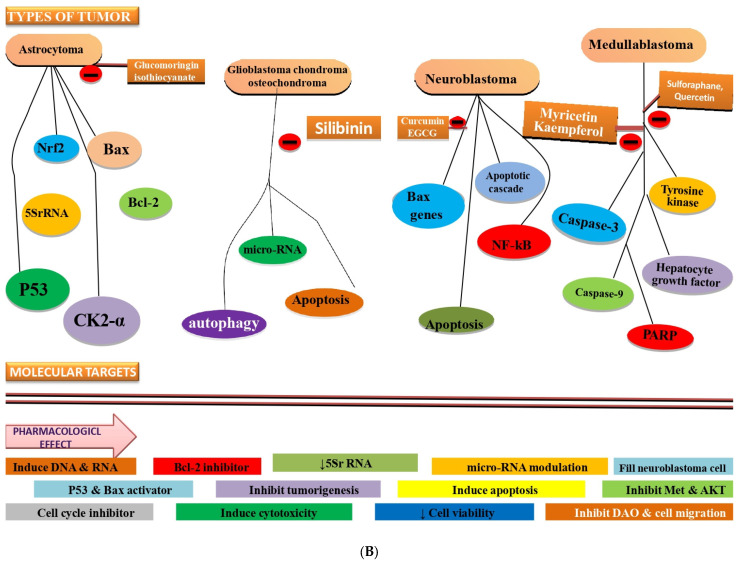

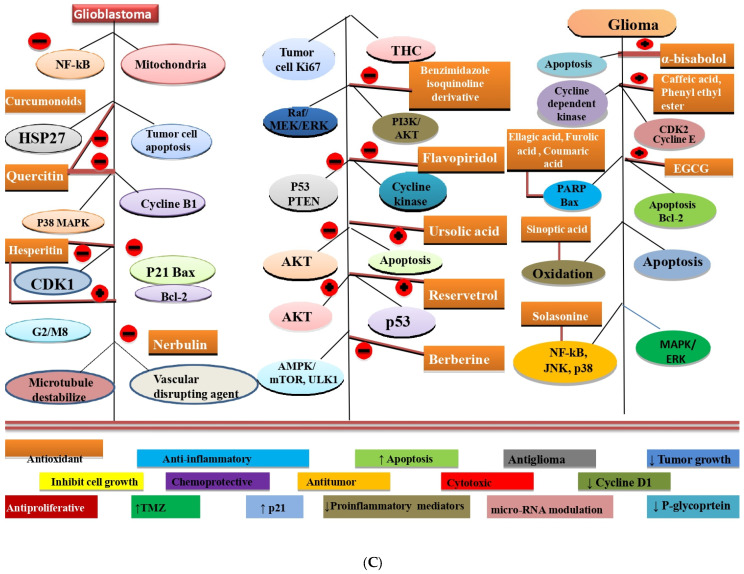
(**A**–**C**) Flowchart showing the specific effect of various phytochemicals on the different types of benign (meningioma, chondroma, pituitary, schwannoma) and malignant tumours (astrocytoma, glioblastoma, chordoma, neuroblastoma, medulloblastoma, osteochondroma) along with their cellular/molecular targets resulting in a plethora of protective mechanisms against tumours.

Despite decades of research, brain tumours are still one of the most lethal type of cancers. Due to the unique cell-intrinsic and microenvironmental features of the brain tissue, these tumours can practically resist all conventional and innovative therapies [41]. Brain tumours have historically been challenging to treat, owing mainly to their biological characteristics [22]. These tumours are hidden behind the blood–brain barrier (BBB), a network of tight junctions and transport proteins that protect fragile neural regions from circulating substances, limiting systemic chemotherapeutic exposure [42]. Furthermore, because of the brain’s distinct developmental, epigenetic, genetic, and other microenvironmental characteristics, these cancers are frequently resistant to novel and traditional therapies [43]. The prevalence of brain tumours exacerbates these difficulties compared to many different types of cancer, which restricts pharmaceutical industry investment and interest [44]. Despite significant advances in cancer treatment, chemotherapy and radiotherapy remain the primary cancer therapy modalities [45]. However, several side effects such as nephrotoxicity, cardiac cytotoxicity, myelosuppression, hepatotoxicity, mucositis, gastrointestinal toxicity, neurotoxicity, and alopecia have a serious impact on the patient’s life [16]. Since many individuals have a desire to live a healthy and natural lifestyle, dietary and plant-derived phytochemicals have gained recognition in recent years [46]. Plants and plant-derived products represent a new option because they are safer, simpler, more environmentally friendly, faster, less toxic, and less expensive [47]. Carcinogenesis is a multi-step process that involves multiple signalling cascades [48]. Taxol, resveratrol, vincristine, quercetin, vinblastine, tetrandrine, and arteannuin are examples of anticancer medicines that modify the autophagy–apoptosis pathway [49]. Polyphenolic compounds and alkaloids are particularly prevalent in cancer therapy [50]. Polyphenols, due to their antioxidant properties, play a vital role in apoptotic, autophagic, and cytostatic effects, making them potential cancer prevention treatments [51] such as taxanes(docetaxel, paclitaxel (PTX)), vinca alkaloids, vinblastine (vinorelbine, vincristine (VCR), vindesine), anthracyclines (doxorubicin, epirubicin, daunorubicin, idarubicin), podophyllotoxin and derivatives (etoposide (ETP), teniposide), andcamptothecin (CPT) [52]. Furthermore, natural products or derivatives account for half of all anti-cancer drugs approved worldwide, and they were developed utilising knowledge gleaned from natural small molecules or macromolecules. By activating caspase-3 and blocking anti-apoptotic proteins, including Bcl-2 and Bcl-xl, quercetin has been shown to promote ROS-stimulated apoptosis and autophagy in various cancers [53,54]. It also reduces apoptosis and intervertebral disc degeneration by activating autophagy through SIRT [55]. Curcumin promotes TRAIL-induced apoptosis via ROS-mediated DR5 overexpression and induces autophagy in cancer cells via the ROS-ERK1/2-p38 MAPK signalling pathway [56,57,58]. Resveratrol has also been shown to be beneficial to health [59] because it triggers ROS-dependent caspases and Bax/caspase-3 in cancer cells, resulting in the destruction of these malignant cells [49,60,61,62]. Despite the availability of advanced cancer treatment options, there is no specific method for completely curing cancer patients [63]. Consequently, anticancer drug development and delivery methods can be further explored by researchers. A wealth of mechanistic data exists on how phytochemicals derived from food with promising chemopreventive properties interfere with tumour development and progression [64]. Several of the mechanisms of the action exerted by these agents, such as regulating oncogenic kinases or cellcycle regulatory molecules, are similar to those used by molecularly targeted chemotherapeutic agents [65]. The anticancer efficacy of these phytochemicals has been studied in vitro and in vivo. Numerous additional mechanisms for slowing carcinogenesis have been reported, including suppression of cancer cell survival and proliferation, tumour invasiveness and angiogenesis [66,67], and free radical scavenging [58]. The phytochemicals act on a wide range of molecular targets and signal transduction pathways, including membrane receptors [68], kinases [69], downstream tumour activator or suppressor proteins [70], transcription factors [71], cyclins, microRNAs (miRNAs) [72], and caspases [66]. Herein, natural products or phytochemicals are the safest and most convenient way to interact with molecular targets involved in brain tumours (Figure 2).

## 3. Limitations in the Use of Phytochemicals/Natural Products for Brain Tumours

Much research has been focused on developing phytochemicals as cancer therapeutic agents over the last few decades. There are still some challenges to their widespread use as medicines [73]. Their several characteristics include a high hepatic disposition, low solubility, low cell penetration, and a narrow therapeutic index. Rapid clearance or absorption by normal tissues, combined with a broad tissue distribution, can result in ineffective drug accumulation at target tumour sites and accidental drug exposure in normal tissues [74]. Phytochemicals are secondary plant metabolites beneficial for disease treatment and human health, but they are large and polar compounds. As a result, they have difficulty crossing the blood–brain barrier (BBB), endothelial blood vessel lining, gastrointestinal tract, and mucosa [75].

Additionally, their absorption is limited by enzymatic degradation in the gastrointestinal tract. Encapsulating or conjugating these drugs with nanocarriers improves gastrointestinal stability, absorption rate, and dispersion [76]. Numerous phytochemicals are used in various therapeutic applications, including phytotherapy, aromatherapy, and gemmotherapy [77]. Effective delivery mechanisms, such as nanoengineered formulations, are required to reap the full benefits of these phytochemicals [75]. Nano-delivery methods can increase the solubility and stability of phytochemicals while also increasing the total blood circulation [78]. Phytochemicals could be prevented from interacting with the biological environment prematurely due to their strong differential absorption ability, increased penetration, and retention qualities in target tissues, resulting in lower toxicity and good dosage optimisation options. Additionally, these advanced delivery systems employ targeted distribution techniques [74,79]. Furthermore, as therapeutic agents, phytochemicals may have unfavourable pharmacokinetics, such as a short elimination half-life and a high clearance rate. Moreover, the emergence of multi-mechanism drug resistance impedes the clinical application of phytochemicals as cancer therapeutic agents [80].

On the other hand, cancer cells can develop resistance to multiple anticancer agents over time, resulting in therapeutic failure [81]. Nanomedicines may overcome phytochemical limitations and associated health concerns by increasing bioavailability, selectively targeting tumour cells and tissue but not normal cells, increasing solubility, increasing cellular absorption, lowering phytochemical doses, and maintaining relatively constant therapeutic phytochemical concentrations over time [82]. Additionally, nanomedicines’ multifunctional nature, excellent blood stability, low interaction with synthetic drugs, and enhanced antitumor activity may be beneficial [83]. The primary mechanism by which nanomedicines target tumours is the enhanced permeability and retention (EPR) effect. Most solid tumours cause inadequate lymphatic drainage and blood vessel development [84]. The EPR effect facilitates the “leaking” of macromolecules and nanomedicines preferentially from blood vessels surrounding a tumour [85]. Most phytochemicals are low-molecular-weight compounds that are rapidly cleared in vivo and distributed widely throughout normal organs and tissues [86,87]. The delivery of phytochemicals via nanocarriers is a more recent method for resolving MDR. Modifications to the biophysical interactions of nanomedicines with lipid components of cancer cell membranes result in an improved phytochemical distribution to target tissues and drug resistance control [88,89]. Nanotechnology-based phytochemical delivery has proven to be an efficient tool for resolving compound delivery problems such as stability, oral bioavailability, and solubility. 

Indeed, nano-based delivery of chemotherapy agents, phytochemicals, and other interesting compounds provides novel distribution strategies such as increased bioavailability, facilitated transport across biological barriers, environmental degradation, protective measures against natural compounds, targeted delivery, and controlled release [90,91]. Recent developments in therapeutic potential through nanomedicines have received considerable attention due to improved phytochemical delivery to tumour and cancer cells [59,92]. Protecting the trapped medicinal drug from degradation, reducing toxicity to normal cells, modifying the pharmacokinetics and tissue distribution profile to optimise drug distribution in tumour cells, and reducing clinical formulation side effects by increasing cellular uptake and improving solubility are considered to be the potential benefits of targeted drug delivery systems in oncology [93]. Phytochemicals’ physicochemical properties and anti-cancer efficacy have been enhanced by applying a wide selection of highly potent nanomedicines [94]. Nanotechnology is receiving global recognition as a vital aspect of biomedical science that focuses on cancer diagnosis [95]. Nanomedicines can effectively adsorb phytochemicals and enhance active, passive, or tissue-specific chemotherapeutics delivery due to their high volume-to-volume surface area ratio, nano-size, surface reactivity, and optical activity [96,97].

Additionally, by delivering the active ingredient to the target location in a controlled and sustained manner, these nanoformulations reduce systemic toxicity while increasing bioavailability [40]. Polymeric nanoparticles, liposomes, polymeric micelles, and nanodispersions are all effective nanocarriers used extensively worldwide [97]. Vincristine sulphate liposome, doxorubicin hydrochloride liposome, irinotecan hydrochloride liposome, paclitaxel nanodispersion injection concentrate, docetaxel nanoparticles, and paclitaxel albumin stabilised nanoparticle formulation have all been approved by the FDA for cancer therapeutics [98]. This review discusses the drawbacks of phytochemicals and their alternatives, including nanocarriers that deliver bioactive compounds directly to tissue targets with increased stability and bioavailability, thereby increasing therapeutic value while avoiding toxicity. Future research should elucidate the structural changes in nanocarriers during digestion and absorption, the discrepancy between in vitro and in vivo digestion simulations, and the effect on phytochemical metabolism. 

## 4. Integrating Nanotechnology in Natural Products/Phytochemical-Based Therapy for Brain Tumours

**Figure 3:**
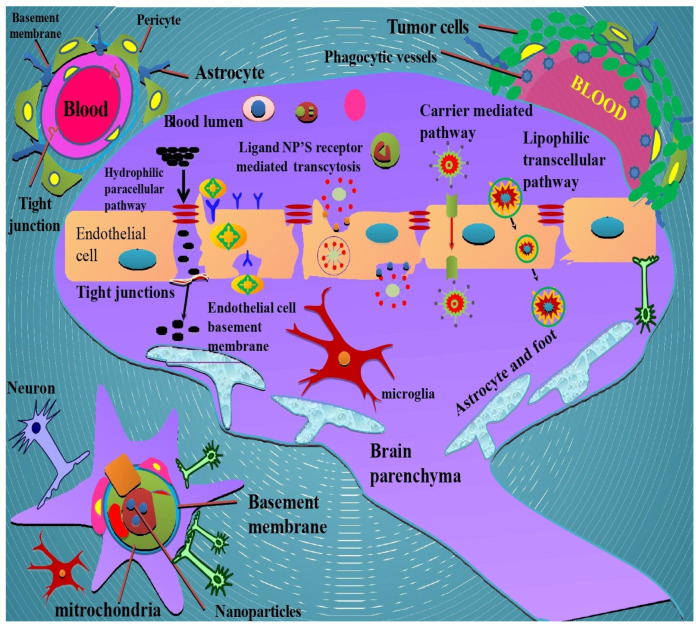
Different mechanisms through which phytochemicals-encapsulated nanoparticles cross the BBB; the distinction between the BBB and BBTB is depicted in this diagram. BBB stands for blood–brain barrier; BBTB stands for blood–brain tumour barrier. Above, a cross-section through the brain; middle, a graphical image of the BBB; below, cellular structure. A network of fine blood vessels runs across the brain. These capillaries transport nutrients and oxygen to the brain. The blood–brain barrier is formed when the walls of these blood vessels are combined. It acts as a physiological barrier between the blood circulation system and the brain in all animals, including humans. Its job is to keep the brain safe from disease-causing agents, toxins, and messenger substances in the bloodstream. The BBB thus functions as a highly selective filter, allowing nutrients for the brain to pass in one direction and metabolic wastes to pass in the other. A series of unique transport processes are required for this supply and removal. The gaps between neurons (nerve cells) in the central nervous system are almost filled by glia or endothelial cells and their processes: (GBM) niche and blood-brain barrier (BBB). To a wide range of molecules, the BBB is selective and restrictive. The cancer stem cells are responsible for treatment resistance in glioblastoma, which comprises heterogeneous cell populations.

The role of nanocarriers in cancer is inevitable as it plays a significant part in both visualization and therapy [26]. Scientists have become increasingly interested in phytochemical encapsulation or conjugation with nanocarriers for delivery to specific areas [99]. Phytochemicals are secondary plant metabolites that have been studied for their potential health and disease-fighting properties. However, transferring these compounds is difficult due to their size and polarity [75]. The primary application of these nanocarriers is to advance cancer chemotherapy. The hydrophobic nature of the anticancer drugs is the possible need for unique target capabilities [26]. Improved permeability is the impact of and retention of cancer nanocarriers, which is considered the value of cancer nanocarriers [100,101]. The use of nanoparticulate drug carriers will resolve several issues associated with drug delivery to cancer cells, including increasing drug solubility, prolonging drug half-life in the blood, increasing drug stability, concentrating drugs at the disease site, and minimizing adverse effects in non-target organs. [102]. The application of nanoparticles in brain tumours has the potential for its treatment since it has been reported that nanoparticles somewhat cross the BBB [103]. Nanoparticles also enhance the selectivity and specificity of therapeutic xenobiotics and other targeting types, allowing them to reach the target site by crossing the BBB, thus enhancing therapeutic efficacy in CNS [26,104]. They are, however, degraded enzymatically in the GIT. As a result, encapsulating or conjugating these compounds with nanocarriers has the potential to improve their bioefficacy by enhancing their absorption rate, dispersion, and gastrointestinal stability [26]. The small intestine’s chemical composition and polarity influence the distribution and absorption of phytochemicals. Small intestine cells are responsible for cellular metabolism as the conversion of these compounds occurs in the hepatic cells, and the pumping of efflux from inactive precursors to active forms is essential for phytochemical absorption [83]. The nanoparticles utilized as carriers are designed to carry phytochemicals to the target region with increased bioefficacy, avoiding the detrimental consequences of these substances being digested in cells and tissues. They are typically constructed at the atomic or molecular level using limited and minimal-sized materials. As a result, they can move more quickly in the human body than larger ones [105,106]. From the standpoint of metabolization, the pathway after oral ingestion is critical in determining where these phytochemicals are recognized and processed by the body as xenobiotics. Phytochemicals will be digested and degraded first in the mouth, then in the stomach and small and large intestines, followed by absorption into the lymph or blood circulation from the GIT. Further dissemination through diffusion or transfer to the circulation of the body occurs. Subsequently, metabolism via biochemical conversion or degradation by body tissues occurs, and excretion occurs through biliary and urinary excretion [83,85,107]. Hepatic cells convert these compounds from inactive precursors to active forms, which is necessary for phytochemical bioavailability [108]. The large intestine microbiota metabolizes unabsorbed phytochemicals from the small intestine, thereby increasing bioefficacy. Additionally, nanoencapsulation technology may be a viable option for delivering bioactive compounds to cells and tissues without the undesirable side effects of metabolization at operating concentrations. Nanoparticles are typically tiny nanostructures formed by atomic or molecular level material engineering. As a result, they can move more quickly than more extensive materials within the human body [106,109]. There are two types of nanoparticles used as nanocarriers: inorganic and organic. The most common inorganic nanocarriers are mesoporous silica, gold/silver, and superparamagnetic iron-oxide nanoparticles (SPIONs) [110]. Inorganic nanoparticle drug delivery is minimal because of the health risks, high toxicity, and poor drug loading efficiency. Liposomes, micelles, niosomes, stable lipid nanoparticles (SLN), archaeosomes, and bilosomes are all organic nanocarriers [111]. Hydrophobic drugs are delivered into the body using lipid-based drug delivery systems. The encapsulating substance prevents the medication from degrading and causing toxicity in the peripheral organs. It increases the drug’s therapeutic index, providing stability, ease of permeability, and successful targeting to the specific site [112,113,114]. Nanotechnology is critical in drug formulation, controlled delivery to the target site, and controlled release [115,116,117,118] (Figure 3).

## 5. A Holistic Strategy for Using Nanoparticles to Fight Brain Tumours

To select a nanoparticle to target the brain, it must have particular properties, such as structure, passive targeting abilities, and surface alteration with active targeting [119]. Polymers, liposomes, inorganic nanoparticles, dendrimers, micelles, and composite nanoparticles are examples of nanoparticles with various properties [120,121]. These are briefly described here:

Physical properties: The surface-to-volume ratio of nanoparticles is the primary attribute that leads to their improved loading functionality [40]. They aid in drug solubility and circulation in the bloodstream and control drug release within tumours. The systemic circulation half-life has been improved by drug molecules or targeted agents such as finasteride [26,59]. Nanoparticles respond to various environmental signals, including temperature and pH, allowing drug release to be tracked. Apart from these features, some nanoparticles have electrical, thermal, and magnetic properties used for diagnostic and therapeutic purposes [122,123,124]. Modifications in nanocarriers’ surfaces help improve certain properties such as surface charge, placement within the bloodstream, hydrophobicity, and even half-life [26]. Various target agents, compounds, and nanoparticles are used to penetrate cells and improve therapeutic efficacy. Surface modification or surface functionalization occurs for BBB-targeting molecules or cell-penetrating peptides [125]. Pegylation, for example, is one such change that improves the half-life of circulating nanoparticles, allowing them to penetrate brain tissues. Nanoparticles can also be combined with targeting agents such as integrins or transmission receptors, expressed on or persisting in the blood of tumour endothelial cells in the tumour’s immediate vicinity [126,127]. As a result, various techniques for targeting brain areas exist, including changing nanoparticle surfaces or combining them with specific receptors and endogenous or exogenous ligands. Recently, for brain cancer/tumour treatment and diagnosis, nanoparticles have emerged as cutting-edge materials. Many nanoparticles for treating brain tumours are in the FDA’s pre-approval stages. Nanoparticles have several ways to pass the BBB. They can cross the BBB either on their own or with a targeted ligand or moiety [128,129,130].

On the basis of the methods used, nanoparticle mechanisms are divided into three categories: the carrier-mediated pathway, the lipophilic transcellular pathway, and the hydrophilic paracellular pathway. Carrier-mediated transport means transporting chemicals through the brain without effluxing back out [131]. The carrier-mediated transport process most likely involves the formation of transient narrow pores caused by binding the specific substrate to the carrier, which allows only the particular substrate molecule to pass [132]. The lipophilic transcellular pathway occurs across the cells. Lipophilicity and molecular weight are the two properties of a substance required for transport across the brain. The higher lipophilicity and the lower molecular weight (<450 Da) of a substance enhance its transport into the brain. In addition, hydrogen bonding is also a crucial consideration in this pathway [132,133]. The hydrophilic paracellular pathway involves the diffusion of substances between cells via intracellular diffusion. It is not saturable or competitive. The tight junctions present on the brain endothelial cells limit paracellular diffusion. Only small hydrophilic molecules can appear to pass through the tight junctions and diffuse through the BBB [132]. By labelling in vivo neuroimaging and exosome monitoring with glucose-coated gold nanoparticles, scientists have established mechanistic structures used by nanoparticles to cross the blood-brain barrier and establish the transit of substances across the BBB [134].

Magnetic particle imaging (MPI) is retrieving popularity due to its submillimetre spatial resolution and high sensitivity [135]. Nanoparticles K16ApoE were highly specific for the Dutch A40 vasculotropic peptide, often present in the cerebral vasculature [136]. Additionally, the nanoparticles developed can be used in hydrophobic therapeutic and imaging agents, enabling the early detection and treatment of pathological changes caused by amyloidosis via magnetic resonance imaging (MRI) [137]. 

Nanomedicine for glioma therapy and diagnostics is an urgent need. The essential aspects of nanoparticles for brain tumours’ treatment are their shape, complicated physicochemical properties, passive and active targeting capabilities, and surface flexibility that can be modified [110,119]. These properties of nanoparticles aid in the identification and treatment of brain tumours and the delivery of therapeutic drugs via the BBB [75].

## 6. Phytochemical Nature and Their Delivery Challenges

Phytochemicals have a wide range of functional groups, polarity, and molecular weights, which affect their solubility and chemical stability [138]. Ideal conditions in delivery vehicles, processing activities, and storage conditions are required to minimise phytochemical loss. If the compounds are degradable, they should be kept away from acidic or alkaline environments [139]. Similarly, if the photochemical deterioration is caused by warmth or light, the pre-proof processing procedure or packaging materials may be designed to avoid these conditions. The development of lipid-based delivery technologies such as nanostructure liposomes and nanoemulsions can improve solubility [140]. When incorporated into liposomes, specific anticancer drugs, such as vinorelbine and curcumin, have increased circulation half-life and fewer adverse effects [141,142]. By allowing for progressive distribution to the target region, the nanoencapsulation approach extends the shelf life of bioactive substances [143,144]. Additionally, the combination of nanocarriers and bioactive phytochemical formulations leads to increased GIT retention, which increases absorption and bioavailability [145,146,147]. The knowledge of cellular absorption and efflux mechanisms, particle form and content, and polarity is essential for the efficient design of nanocarriers to deliver phytochemicals.

## 7. Phytochemical Nanocarriers

These nanocarriers are designed to carry phytochemicals more efficiently to the target site. Nanocarriers come in a variety of shapes and sizes. Depending on the carrier substance, hydrophilic and hydrophobic molecules are enclosed. For example, lipid-based nanocarriers transport both polar and non-polar molecules in the aqueous core and membrane. 

### 7.1. Inorganic Nanocarriers

Micelles and inorganic nanoparticles have a similar structure. They possess an inorganic core made of gold, silica, silver, iron oxide, and other elements, while organic polymers or metals can make up their shells [148]. The shell functions as a biomolecule or receptor attachment site, protecting the drug from external physiological changes. Nanoparticles with inorganic compounds have plasmonic and magnetic characteristics [149]. Covalent or ionic contacts and physical absorption can be used to attach drug molecules to nanoparticles. Light or physical stimulation can be used for administration and controlled drug release [53]. FeO nanoparticles coated with violamycin B1 and anthracycline antibiotics were studied for their antiproliferative and cytotoxic effects [150].

### 7.2. Organic Nanocarriers and Lipid-Based Nanocarriers

Hydrophobic medications are delivered into the body using lipid-based drug delivery systems. The encapsulated substance prevents the medicine from degrading and causing toxicity in the peripheral organs. It improves the drug’s therapeutic index, provides stability, eases permeability, and allows for successful site-specific targeting. Because of its bioactive nature, phytochemical coating or loading efficiently decreases toxicity and provides different therapeutic qualities [151]. Nanoencapsulation of nutraceuticals is an essential method for providing food ingredients, nutrients, dietary supplements, and other bioactive products, wherein saponins, flavonoids, alkaloids, isoflavones, organic acids, tannins, and catechins are included. Curcumin, encapsulated in nanoemulsion employing medium-chain triacylglycerols as an oil base and Tween 20 as an emulsifier, was beneficial in reducing rat ear oedema induced by TPA when compared to free curcumin [152]. Many diseases, including cancer and age-related ailments, are caused by the development of reactive oxygen species (ROS). Because phytochemicals have strong antioxidant capabilities, this is one of the most studied applications for phytochemical linked nanoparticles or nanoemulsions. During the fatty stage, oil-in-water nanoemulsions containing olive oil and esterified epigallocatechin gallate (a powerful antioxidant) improved antioxidant activity [153,154]. 

In another study, gold nanoparticles synthesized and stabilized with tea extracts and gum arabic were shown to internalize MCF-7 breast cancer cells and PC-3 prostate cancer cells, demonstrating a non-toxic alternative for AuNPs as a therapeutic diagnostic agent [155].

Stability is crucial in the case of resveratrol, a polyphenol found in a variety of berries and nuts that is utilized as a dietary supplement to treat cardiovascular disease and cancer.

Nano-encapsulated phytochemical forms in nanospheres, liposomes, stable lipid nanoparticles, and polymeric lipid-core nanocapsules increased trans-water resveratrol’s solubility and boosted its photo-stability under UV exposure [156]. Encapsulation or conjugation of these medications with nanocarriers can improve their bioefficacy by modifying their gastrointestinal stability, absorption rate, and dispersion. As a result, nanoencapsulation technology could be employed to distribute bioactive constituents differently. Nanocarriers help to promote efficacy and reduce toxicity by transporting bioactive chemicals directly to the target organ with improved bioavailability and stability. The impact of nanocarriers, their structural changes during digestion and absorption, and the discrepancy between in vitro and in vivo digestion simulations are essential topics of concern to study more in the future.

**Research-Based Detailed Data (Pre-Clinical and Clinical Studies) of Phytochemicals/Natural Products in the Prevention of Brain Tumours (Benign and Malignant) via Modulating Particular Cellular/Molecular Signalling: Table 1; Table 2**.

## 8. Conclusions and Future Perspectives

The most invasive forms of cancers are known to be brain cancers, and a vast range of approaches are being tested to treat and overcome them. Nanoparticles have gained considerable interest in the treatment and diagnosis of brain tumours. Many nanoparticles are in the FDA pre-approval stages to treat brain cancers. This review discusses brain tumours, phytochemicals, their pathways, molecular targets, brain tumour phytochemical limits, and the integration of existing research conditions of cancer treatment methods for phytochemical nano-drug delivery. In different nervous system tumours, both benign and malignant, the effect of phytochemicals on molecular targets is highlighted. In addition, the properties of nanoparticles to treat brain cancers effectively, the different mechanisms used to enter targeted tumour sites, and the various forms of nanoparticles used to treat brain tumours have also been considered a part of the discussion. So, to use phytochemicals combined with nanotechnology for potential clinical and biomedical applications in brain tumours, the review concludes with a summary of the future perspectives and further obstacles to be faced.

The potential for success in biomedical applications has expanded since the introduction of nanotechnology. Phytonanomedicine is used as a diagnostic as well as a therapeutic tool. Nanotechnology is being used to treat various serious disorders, including brain cancer, one of the major causes of death and morbidity worldwide. Numerous studies have cited nanoparticles have been used as an excellent solution in theranostic applications of brain cancer; for instance, the in vivo PTT of mice carrying subcutaneous U87 xenograft tumours contains a significant number of nanoparticles, including the PBS group, the nanoparticle group, the laser group, and the laser and nanoparticle classes [187]. Nanomedicines, a potential therapy for brain cancer, has received FDA approval and are currently being tested in clinical trials for disease treatment [188]. The surface functionalization of nanoparticles is a critical factor in optimizing their properties. As a result, they can cross the BBB more quickly to reach tumour locations [189]. The second step is to use polymer-based lipids and nanoparticles such as polymers, dendrimers, micelles, and liposomes more frequently because they are less hazardous and can endure a wide pH and temperature range. Some are FDA approved [59,110]. The next phase is to develop other protein-based and inorganic nanoparticles with high vector potential. Because of their electrical, thermal, and fluorescent properties, inorganic nanoparticles have distinct structural properties and can be used to treat and diagnose brain tumours [190]. As a result, they may be used as contrast agents in diagnostics and as targeting agents in therapeutics.

Furthermore, to cross the BBB, the size of the nanoparticles must be small enough. The stability of the nanoparticles is maximized to remain within the tumours and increase their therapeutic time. The second problem concerns the toxic effects of nanoparticles, which need to be taken into account for long-term use [191]. Because of their unique structures or surface modifications, most nanoparticles are toxic. Their synthesis method, manufacturing costs, development challenges, and ultimate entry into the worldwide market for clinical trials are the third issues that need to be addressed. The fourth problem is that the containers or processing units used during their long manufacturing period must be washed so as not to obstruct the nanoparticles’ stability and properties [192]. Finally, it is critical to assess the retention period of nanoparticles alongside normal organs within tumours and use a simple method of nanoparticle clearance [102]. Pharmacokinetics and pharmacodynamics tests, which are toxicity monitors, are used further to assess the safety of nanoparticles inside the body. Various immunological assays and tests evaluate the immunological response produced by nanoparticles within the body and their possible use in therapeutics and diagnostics.

## Figures and Tables

**Table 1:** Summarised nano-based phytochemicals/natural products in benign brain tumours.

S. No.	Brain Tumour Type	Phytochemicals	Target	Pharmacological	Study Type	Dose and Route	Duration of Study	Key Finding	References
	(Benign)	Natural Products	Involvement	Action	(Pre-Clinical/Clinical)				
**1**	Chordoma	17-Allylamino-geldanamycin Bortezomib Digoxin Vincristine	Heat shock protein 90, U-CH1, IC50, µM inhibition U-CH2, IC50, µM inhibition CCL4, IC50, µM Proteasome U-CH1, IC50, µM Na^+^/K^+^ ATPase Microtubule	Cytotoxicity Caspase inhibition	Clinical trials	10 to 370 nM EC50 of 7.08 μM 9.73 μM 20 nM	16 h 24 h 48 h	Bortezomib combined with topoisomerase I and II inhibitors improved therapeutic efficacy inU-CH2 and patient-derived primary cultures	[157]
**2**	Human meningioma	AR42	Histone deacetylase	Histone deacetylase inhibitor	Cell culture (in vitro)	0.75–3.0 μM	1–2 days	Inhibiting transcription/translation of Akt gene Destabilising Akt protein Inhibiting histone deacetylase	[158]
**3**	Pituitary tumours	Genistein	Cell cycle CDKN1A Bcl2 mRNA mKi67 mRNA pH3 immunostaining	Inhibits proliferation a Induces senescence	Male and female neonatal CD-1 mice (in vitro)	0.06 μM to 36 μM	10 days	↓ Cell proliferation ↓ Bcl2 mRNA levels ↓ mKi67 mRNA Cell cycle impairment ↓ mRNA of pH3 and Ccnb1 immunostaining	[159]
**4**	Pituitary tumours	R-equol	MAPKs Nongenomic signalling pathway	R-eq suppressed 1nM E2-activated ERK, JNK, and p38, as well as cell proliferation R-eq augmented intracellular calcium levels and caused prolactin release; in contrast to E2, increased cell proliferation, as estrogens that activate ERK often do	GH3/B6/F10 cells (Cell culture)	10^–16^ to 10^–7^ M	3 days	↑ MAPKs ↑ Intracellular calcium Activated Gαi, ↑ PRL release in time frames consistent with rapid nongenomic signalling pathway actions	[160]
**5**	Schwannoma	Curcumin, NDGA, EGCG	Amyloid fibril formation TTR amyloid fibrils	Curcumin strongly suppressed TTR amyloid fibril formation NDGA slightly reduced TTR aggregation EGCG maintained most of the protein in a non-aggregated soluble form	Either sex rat schwannoma (RN22) cell line (in vitro)	Curcumin; 0.1 and 0.02 NDGA; 0.1 and 0.02 phytochemicals (5 μL of a 10 mM solution)	16 days	↓ Extracellular TTR oligomeric induced toxicity Disaggregated amyloid fibrils ↓ levels and size of TTR fibrils	[161]

Phytochemicals and their complete data, including dose, route, effect on specific molecular targets, pharmacological effect, and key findings of various research from preclinical (in vivo, in vitro, ex vivo) studies and clinical trials involved in various benign brain tumours, are listed here.

**Table 2:** Summarised nano-based phytochemicals/natural products in malignant brain tumours.

S. No.	Brain Tumour	Phytochemicals/Natural Products	Target Involvement	Pharmacological Action	Study Type	Dose and Route	Duration of Study	Key Finding	References
	(Malignant)				(Pre-Clinical/				
					Clinical)				
1	Astrocytoma grade IV	Glucomoringin isothiocyanate	p53 Bax, Bcl-2 Nrf2 transcription factor CK2 alpha 5S rRNA	Inhibit tumorigenesis	Human brain astrocytoma cell line (CCF-STTG1) (in vitro)	2–40 μM	24 h	Antitumor efficacy of moringin ↓ 5S rRNA Induce DNA andRNA fragmentation in CCF-SSTG1 cells p53 and Bax activation Bcl-2 inhibition	[162]
2	Glioma	α-Bisabolol	Mitochondrial Apoptosis intrinsic pathway Apoptosis extrinsic pathway	Induces apoptosis in glioma cells and inhibits tumour cells	Human andrat glioma cell lines(in vitro)	10 μM	24 h	Cytotoxic effect of α bisabolol Apoptosis induction Release of cyt-c Inhibit cell growth	[163]
		Caffeic acid phenethyl ester	Cyclin-dependent kinase CDK2 cyclin E pRb	Antitumor Inhibit C6 glioma cells, Increased G0/G1 phase	Male Wistar rats, C6-glioma cells and 4–6 weeks old BALB/c-nu female nude mice, (18–20 g) (in vitro and in vivo)	50 μM (Cell culture) 1–10 mg/kg (Intraperitoneal)	36 h	Inhibited C6 glioma cells ↓ Number of mitotic cells ↓ PCNA in C6 glioma	[164]
		EGCG	P-glycoprotein Apoptosis p-Akt Bcl-2 PARP	Antitumor, PI3K inhibitor	Rat glioma cell line C6 and human glioblastoma cell lines U87, U251, SHG-44 (in vitro)	0–200 μM	24 h	↓ P-glycoprotein ↓ p-Akt Inhibit cell viability	[165]
		Curcumin	Wnt signalling pathway	Anti-tumour, induce apoptosis, increase the differentiation rate of neurons in neural stem cells	14.5-day-old pregnant SD rats (in vitro)	500 nmol/L	72 h	Neuroprotective effect Changes in the downstream wnt signalling pathways	[166]
		Root extracts of Leonurus sibiricus (ferulic acid; caffeic acid; ellagic acid; chlorogenic acid; p-coumaric acid; verbascoside)	S- and G2/M-phase cell cycle Bax/Bcl-2 p53	Anti-cancer activity, cytotoxic effect	56-year-old patient (human glioblastoma primary cell line) (in vitro)	0.85 mg/mL 1.25 mg/mL 2 mg/mL 2.4 mg/mL	24 h	Exhibits anti-cancer activity Regulation of genes involved in apoptosis.	[167]
		Sinapic acid (SA)	BBB Oxidative agent Apoptosis marker	Potential glioma treatment to reduce neurotoxicity	C6 rat glioma cells and RBMEC (in vitro)	0–200 μM	50 days	↑ BBB-permeable Induces strong apoptosis	[168]
		Solasonine	NF-κBsignalling pathway JNK and p38/MAPK ERK/MAPK	Inhibition of NF-κBsignalling pathway Anti-inflammatory Inhibition of JNK and p38 phosphorylation and ERK/MAPK phosphorylation	U87 MG cells (in vitro)	0–8 μM	30 days	Inhibits glioma growth Suppression of MAPK ↓ Cell proliferation ↓ Proinflammatory mediators	[169]
3	Glioblastoma, Chondroma, Osteochondroma	Silibinin	Autophagy Cell cycle microRNAs Apoptotic marker	Cell cycle inhibitor, autophagy modulator, apoptotic inducer	Xenograft mice model	100 μg/mL 200 mM	48 h	microRNAs modulation Cell cycle inhibition Induces apoptosis	[170]
4	Glioblastoma	Curcuminoids	NF-κB pathway Mitochondria Caspase-dependent pathway	Antioxidant Anti-inflammatory Anti-proliferative Potent chemo-preventive action	Human brain GBM 8401 cells (in vitro)	0 μM 12.5 μM 25 μM 50 μM	48 h	Inhibits cell proliferation Activation of apoptosis	[171]
		Quercetin	Hsp27 Tumour cell apoptosis	Anti-tumour Protect normal cells Facilitate tumour cell apoptosis	U251 and U87 human glioblastoma cell line and MTT assay (in vitro)	30 μmol/L	48 h	↓ Proliferation and viability of glioma cells Inhibits Hsp27 expression	[172]
		Hesperetin	p38 MAPK Cyclin B1 CDK1 p21 G2/M 8	Antioxidant Anti-inflammatory Anticancer	Human GBM cell lines U-251 and U-87 (in vitro)	0 µM 200 µM 400 µM 600 µM 800 µM	48 h	Apoptotic cell death ↓ Bcl-2 ↓ Bax Inhibits p38 MAPK Promotes cellcycle arrest	[173]
		Punicalagin Cyanidin-3-glucoside	Glioma cells	Cytotoxic effects on glioma cell lines Anti-glioma	SVG-p12 and U87-MG cells (in vitro)	46 μM 49μM	3 days	Potential treatment for glioblastoma	[174]
		Verubulin	Microtubule destabiliser Vascular disrupting agent		Male and female 23–77 years old GBM patients	2.1 mg/m^2^ 3.3 mg/m^2^ (Intravenous infusion)	28 days	Verubulin has no single-agent activity against recurrent GB	[175]
		Tetrahydrocannabinol (THC)	Tumour-cell Ki67	Antitumoral, inhibiting tumour-cell proliferation	Nine patients with GBM (tumour cell cultures)	0.5 µM 1 µM 2 µM 2.5 µM	32 weeks	Inhibited tumourcell proliferation ↓ tumour cell Ki67	[176]
		Benzimidazoleisoquinolinone derivatives	Raf/MEK/ERK pathway PI3K/AKT pathway	Anticancer or antitumor	Human U87 and LN229 cell lines (in vitro)	0 µM 6.25 µM 12.5 µM 25 µM 50 µM 100 µM	14 days	↑P21 and P53 ↓ Cyclin A and E Inducesapoptosis	[177]
		Flavopiridol	p53 EGFR PTEN Cyclin-dependent kinase	Antiproliferative and apoptotic effects	U87MG, T98G, and U118MG cell line (in vitro)	150 nM-10 µM	72 h	↓ Cyclin D1 activities ↓ c-Myc activities ↓ p53 protein activities ↑ p27KIP1	[178]
		Ursolic acid (UA)	AKT signalling pathways Apoptosis	Akt phosphorylation Increased sub-G1 fraction and induced apoptotic death	C6 rat glioma cells (in vitro) 8–9-week-old male Wistar rats (invitro)	In-vitro dose; 5 µM 7.5 µM 10 µM 15 µM or 20 µM In-vivo dose; 5 mg/kg/day 15 mg/kg/day (Intraperitoneal)	15 days (In-vitro) 10 days (In-vivo)	↓ Tumor growth ↑ Efficacy of TMZ by UA Potential therapeutic effects	[179]
		Resveratrol	AKT signalling pathway p53	Inhibits cell proliferation, sphere-forming ability, and invasion	Patients’ GSCs, U87 glioma cell line and 5–6-week-old female BALB/c Nude mice (in vitro and in vivo)	100 mg 125 mg 25mg/kg/day 50mg/kg/day (oral)	21 days	Inhibited proliferation Inhibited GSC cell lines Blocked U87 glioma	[180]
		Berberine (BBR)	AMPK/mTOR/ULK1 pathway	Reduces Tumour growth, causes autophagy, has potent antitumor effects, and causes inhibition of EGFR	Human glioma cell lines (U251 and U87) and 4-week-old male Athymic mice (20–30 gm)	In-vitro dose; 50 µM 100 µM 150 µM 200 µM 250 µM Invivo dose; 50 mg/kg/day	18 day	↓Tumour growth Potential clinical benefits for autophagy Inhibits the AMPK/mTOR/ULK1 pathway	[181]
5	Brain neoplasm	β-Carotene	Reactive oxygen species	Antioxidant	1273 men and 1293 placebo (case–control study)	50 mg Alternate days	12 years (Trial)	Beta carotene produced neither benefit nor harm	[182]
6	Neuroblastoma	Curcumin EGCG	Brain-expressed X-linked (Bex) genes Induces apoptosis NF-κB Apoptotic cascade	Induced all endogenous Bex genes OGmiRs TSmiRs	N2a neuroblastoma cell (in vitro) Human malignant neuroblastoma SK-N-BE2 and IMR-32 cells (in vitro)	10 μM 25 μM 50 μM 25 μM 50 μM 100 μM	2 days 72 h	Kills N2a neuroblastoma cells Induce apoptosis ↓ OGmiRs ↑ TSmiRs ↓ Cell viability	[183]
									[184]
7	Medulloblastoma (MBL)	Sulforaphane	Caspase-3 and -9 activities Cleavage of PARP and vimentin	Cell death by apoptosis DNA fragmentation and chromatin condensation	HT-29 and Caki-1 cell lines and U-87 MG cell line (in vitro)	10 μM 20 μM	72 h	Inducescytoxicity Novel inducer of MBL cell apoptosis Chemo preventive agents	[185]
		Quercetin Kaempferol Myricetin	Hepatocyte growth factor Tyrosine kinase Met	Diminished HGF-mediated Akt activation and tyrosine kinase receptor Met	Human DAOY medulloblastoma cell line (in vitro)	0 μM/L 5 μM/L 10 μM/L 15 μM/L 20 μM/L Kaempferol, quercetin, and myricetin	3 h	Inhibits DAOY cell migration Inhibits Met and Akt Prevents invasion and metastasis	[186]

Phytochemicals and their complete data, including dose, route, effect on specific molecular targets, pharmacological effect, key findings of various research from preclinical (in vivo, in vitro, ex vivo) studies, and clinical trials involved in various malignant brain tumours, are listed here. Symbols: (↑) increase; (↓) decrease. +—positive effect; −—negative/inhibitory effect.

## Data Availability

Not applicable.

## Abbreviations

AKT	protein kinase B (PKB)
AuNPs	gold nanoparticles
BAX	bcl-2-like protein 4
BBB	blood–brain barrier
bcl-2	B-cell lymphoma 2
CMT	Carrier-mediated transport
CD31	cluster of differentiation 31
CDK	cyclin-dependent kinases
CPT	camptothecin
Cyclin B1	regulatory protein
EGCG	epigallocatechin gallate
EPR	electro paramagnetic resonance
ERK	extracellular-signal-regulated kinase
ETP	etoposide
FDA	Food and Drug Administration
GBM	glioblastoma multiforme
GTI	gastrointestinal tract
HSP	heat shock protein
JNK	c-Jun N-terminal kinase
K16ApoE	transporter
MAPK	mitogen-activated protein kinase
MCF7	breast cancer cell line isolated in 1970
MKKS	McKusick–Kaufman/Bardet–Biedl syndromes putative chaperonin
MPI	magnetic particle imaging
mTOR	mechanistic target of rapamycin (mTOR),
NIR	near-infrared radiation
NB	neuroblastoma
NDGA	nordihydroguaiaretic acid
NF-κB	(nuclear factor kappa light chain enhancer of activated B cells
Nrf2	nuclear factor erythroid 2-related factor 2
PARP	poly-ADP ribose polymerase
PEITC	phen ethyl isothiocyanate
PC3	prostate cancer cell type3
PTT	partial thromboplastin time
PTX	paclitaxel
p13k	phosphoinositide 3-kinase
p-38	mitogen-activated protein kinases
p53 orTP53 or tumour protein	
ROS	reactive oxygen species
SLN	stable lipid nanoparticles
SPIONs	super paramagnetic iron-oxide nanoparticles
THC	tetrahydrocannabinol
Tk	tyrosine kinase
TPA	tissue plasminogen activator
TxA2	thromboxane A2
VCR	vincristine
WNT	wingless-related integration site
